# UNLOCKING PAIN MANAGEMENT FOR PEOPLE LIVING WITH DEMENTIA: AN INTERVENTION TO BE TAILORED

**DOI:** 10.1093/geroni/igad104.3746

**Published:** 2023-12-21

**Authors:** Annalisa Na, Justine Sefcik, Amy Kwok, Molly Drazon, Laura Gitlin

**Affiliations:** Drexel University, Philadelphia, Pennsylvania, United States; Drexel University College of Nursing and Health Professions, Philadelphia, Pennsylvania, United States; Drexel University, Philadelphia, Pennsylvania, United States; Drexel University, Philadelphia, Pennsylvania, United States; Drexel University, Philadelphia, Pennsylvania, United States

## Abstract

Effective pain management for persons living with dementia (PLWD) is critical to quality of life and could be achieve through rehabilitative care. However, it’s not well understood how physical therapists (PTs) and occupational therapists (OTs) develop and deliver treatment plans for PLWD, as most pain studies and evidence-based guidelines explicitly exclude those with cognitive impairment. In this qualitative descriptive study, we explored clinicians’ perceptions of pain management in PLWD as recommended by available guidelines. Semi-structured virtual interviews of 6 PTs and 4 OTs from 4 outpatient facilities were all female, with an average age of 36.7 (SD 8.78) years and an average clinical experience of 7.01 (SD 4.81) years. We analyzed transcripts using conventional content analysis and identified a consistent theme that rehabilitative-based interventions for pain management among PLWD need to be “carefully tailored.” Clinicians explained that the plan of care they designed for PLWD need to be tailored to the patient’s underlying pain mechanism, physical function, and comorbidities that can affect their participation in therapy, to avoid activities that exacerbate pain symptoms, and take into consideration the extent of memory loss that the person is experiencing and their preferences. This study highlights the important factors practicing clinicians take into consideration when developing rehabilitative programs to manage pain among PLWD. These findings can guide the updating of current pain management guidelines so they are more inclusive and tailored to PLWD.

